# Mesenchymal stem cell–derived exosome delivery of let-7a-5p enhances macrophage efferocytosis via Arid3a/Mertk axis in acute-on-chronic liver failure

**DOI:** 10.1093/stcltm/szaf058

**Published:** 2025-12-02

**Authors:** Junyi Wang, Zhihui Li, Zhouhan Wang, Wei Liang, Shibo Meng, Junfeng Chen, Jialei Wang, Jing Zhang, Bingliang Lin

**Affiliations:** Department of Infectious Diseases, The Third Affiliated Hospital of Sun Yat-sen University, Guangzhou 510630, China; Guangdong Key Laboratory of Liver Disease Research, The Third Affiliated Hospital of Sun Yat-sen University, Guangzhou 510630, China; Department of Infectious Diseases, The Third Affiliated Hospital of Sun Yat-sen University, Guangzhou 510630, China; Guangdong Key Laboratory of Liver Disease Research, The Third Affiliated Hospital of Sun Yat-sen University, Guangzhou 510630, China; Department of Infectious Diseases, The Third Affiliated Hospital of Sun Yat-sen University, Guangzhou 510630, China; Guangdong Key Laboratory of Liver Disease Research, The Third Affiliated Hospital of Sun Yat-sen University, Guangzhou 510630, China; Department of Infectious Diseases, The Third Affiliated Hospital of Sun Yat-sen University, Guangzhou 510630, China; Guangdong Key Laboratory of Liver Disease Research, The Third Affiliated Hospital of Sun Yat-sen University, Guangzhou 510630, China; Department of Infectious Diseases, The Third Affiliated Hospital of Sun Yat-sen University, Guangzhou 510630, China; Guangdong Key Laboratory of Liver Disease Research, The Third Affiliated Hospital of Sun Yat-sen University, Guangzhou 510630, China; Department of Infectious Diseases, The Third Affiliated Hospital of Sun Yat-sen University, Guangzhou 510630, China; Guangdong Key Laboratory of Liver Disease Research, The Third Affiliated Hospital of Sun Yat-sen University, Guangzhou 510630, China; Department of Infectious Diseases, The Third Affiliated Hospital of Sun Yat-sen University, Guangzhou 510630, China; Guangdong Key Laboratory of Liver Disease Research, The Third Affiliated Hospital of Sun Yat-sen University, Guangzhou 510630, China; Department of Infectious Diseases, The Third Affiliated Hospital of Sun Yat-sen University, Guangzhou 510630, China; Guangdong Key Laboratory of Liver Disease Research, The Third Affiliated Hospital of Sun Yat-sen University, Guangzhou 510630, China; Department of Infectious Diseases, The Third Affiliated Hospital of Sun Yat-sen University, Guangzhou 510630, China; Guangdong Key Laboratory of Liver Disease Research, The Third Affiliated Hospital of Sun Yat-sen University, Guangzhou 510630, China; Key Laboratory of Tropical Disease Control (Sun Yat-sen University), Ministry of Education, Guangzhou 510080, China

**Keywords:** acute-on-chronic liver failure, efferocytosis, exosome, let-7a-5p, macrophage, mesenchymal stem cell, Mertk

## Abstract

**Background:**

Acute-on-chronic liver failure (ACLF) is a severe clinical syndrome with a high mortality rate and limited therapeutic options. Macrophage efferocytosis plays an essential role in maintaining tissue homeostasis, and its dysfunction may be associated with the pathogenesis of ACLF. We previously found that mesenchymal stem cell (MSC) treatment in ACLF mice promoted macrophage M2 polarization and elevated the efferocytosis-related protein Mertk, but the underlying mechanisms remained unclear.

**Methods:**

The role of efferocytosis was investigated in liver tissues from ACLF patients and an ACLF mouse model treated with MSC-derived exosomes (MSC-Exos). *In vitro* experiments utilizing lipopolysaccharide-induced M1 macrophages were conducted to dissect the underlying mechanism, targeting the miRNA let-7a-5p. Engineered exosomes (MSC-Exos^let-7a-5p^) were developed via electroporation to validate the therapeutic potential.

**Results:**

Impaired macrophage efferocytosis in liver tissues correlated with poor prognosis in ACLF patients. Treatment with MSC-Exos significantly improved histological morphology, liver function and enhanced efferocytosis in ACLF mice. Mechanistically, MSC-Exos delivered let-7a-5p to M1 macrophages, which downregulated Arid3a and upregulated Mertk expression. Furthermore, engineered MSC-Exos^let-7a-5p^ promoted efferocytosis more effectively than unmodified exosomes.

**Conclusion:**

MSC-Exos enhance macrophage efferocytosis in ACLF via the let-7a-5p/Arid3a/Mertk axis. Engineered MSC-Exos^let-7a-5p^, by boosting this pathway, provide a potential strategy for improving ACLF therapy.

## Graphical abstract

**Graphical Abstract:**
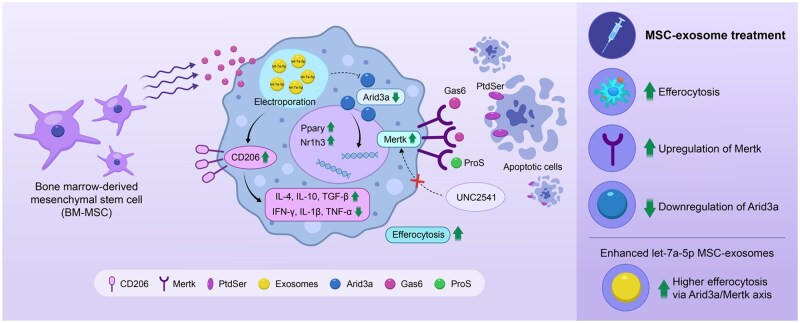

Significance statement ACLF is a globally challenging condition that is linked to impaired macrophage efferocytosis. This study reveals that MSC-Exos deliver microRNA let-7a-5p, which enhances efferocytosis by modulating the Arid3a/Mertk axis. Engineered MSC-Exos loaded with let-7a-5p demonstrated superior therapeutic effects in clearing dead cells compared to natural MSC-Exos. These findings identify let-7a-5p-enriched MSC-Exos as a novel strategy for enhancing macrophage efferocytosis in ACLF, combining targeted molecular intervention with extracellular vesicle-delivery advantages. This approach holds translational potential for developing precision therapies against ACLF progression.

## Introduction

Acute-on-chronic liver failure (ACLF) is the acute decompensation of chronic liver disease, characterized by a strong systemic inflammatory response, the failure of one or more organs, and a high mortality rate within 28 days of onset.^1^ Currently, the only definitive treatment is liver transplantation; however, shortage of donors, expense, and risk of postoperative immune rejection severely hamper widespread application.^2^

Macrophages are generally categorized into two main types: M1 macrophages (pro-inflammatory) and M2 macrophages (anti-inflammatory or reparative).^3^ M1 macrophages participate in inflammatory responses by releasing various pro-inflammatory cytokines, whereas M2 macrophages regulate inflammation and promote the healing of damaged tissues through the secretion of anti-inflammatory cytokines, such as IL-10 and TGF-β, as well as factors that support tissue repair.^4^ In ACLF, the liver upregulates damage-associated molecular pattern (DAMP) receptors in Kupffer cells (KCs, liver-resident macrophages), such as toll-like receptor (TLR) 4, TLR9, and receptor for advanced glycation end products.^5^ Activated KCs secrete pro-inflammatory cytokines, such as TNF-α, reactive oxygen species, and chemokines (chemokine C–C motif ligand 2). These elements amplify pro-inflammatory signals and enhance the recruitment of bone marrow–derived immune cells, predominantly neutrophils and monocytes, to the liver. Monocytes differentiate into mature macrophages and intensify inflammation. Under these conditions, macrophages polarize to the pro-inflammatory M1 phenotype. During this process, macrophages not only act as sensors for DAMPs and pathogen-associated molecular patterns but also serve as effectors that exacerbate inflammation. The release of pro-inflammatory cytokines disrupts immune homeostasis, triggering a “cytokine storm” and inducing systemic inflammatory responses. Consequently, a substantial number of hepatocytes undergo various forms of programmed cell death, including necrosis, apoptosis, pyroptosis, and ferroptosis.^6^ As ACLF progresses, M2 macrophages play a critical role in mitigating inflammatory responses and facilitating liver repair. This strongly indicates that M1 polarization of macrophages is a pivotal factor in the onset and progression of ACLF.

Cell death and efficient clearance of dead cells are fundamental for maintaining tissue homeostasis. The rapid removal of apoptotic cells through macrophage efferocytosis prevents their accumulation and promotes recovery after tissue damage, indicating that dead cells are rarely observed under normal conditions.^7^ During efferocytosis, macrophages release pro-resolution mediators that activate repair-promoting transcriptional programs, facilitating a shift from the pro-inflammatory M1 phenotype to the anti-inflammatory M2 phenotype.^8^ The weakening of macrophage efferocytosis has been observed in various chronic inflammatory diseases.^9^

Mertk is a member of the TAM receptor family and a key membrane receptor on macrophages. Through bridging proteins (eg, Gas6 and S protein), Mertk recognizes the “eat me” signal by binding to phosphatidylserine exposed on the surface of apoptotic cells, thereby initiating phagocytosis.^10^ In our previous study,^11^ we revealed that infusion of bone marrow–derived mesenchymal stem cells (BM-MSCs) into ACLF mice enhanced M2 polarization and increased Mertk expression. These changes reduced inflammatory damage in the liver, thus improving liver function and survival rates. Infusion of BM-MSCs also significantly enhanced survival rates in patients with ACLF.^12^ However, we did not determine whether these effects are directly associated with efferocytosis.

The transcription factor Arid3a is a member of the ARID protein family and plays a crucial role in regulating gene expression.^13^ Arid3a negatively regulates Mertk expression in macrophages, impairing efferocytosis and exacerbating cholestatic liver injury.^14^ However, no studies have examined whether Arid3a remains an upstream regulator of Mertk expression during ACLF and consequently influences efferocytosis under pathological conditions.

The most prominent negative regulators of Arid3a are the let-7 microRNA family.^15^ Previously, we demonstrated that let-7a-5p naturally exists in MSC-Exos and may serve as a mediator of the hepatoprotective autophagic repair undertaken by them.^16^ In the present study, we aimed to verify this finding in ACLF. Our objectives were to determine the role of efferocytosis in ACLF and identify the underlying molecular mechanisms, specifically the involvement of Mertk. Furthermore, we investigated whether MSC-Exos could alleviate the inflammatory response associated with ACLF. Our study contributes important data that could aid in the development of an effective ACLF treatment.

## Methods 

### Human liver tissue samples

Liver samples were acquired from eight patients with ACLF and three healthy controls (HCs). The diagnosis of patients was made according to the chronic liver failure (CLIF) criteria.^17^ ACLF samples were collected during liver transplantation, whereas HC samples were collected from non-lesional liver regions during hemangioma surgery. All patients were evaluated using several clinical prognostic models based on their clinical presentation and serological data, including Model for End-Stage Liver Disease (MELD), CLIF-C ACLF, Child-Pugh, and COSSH-ACLF scores. For further analysis, patients were split into groups based on their CLIF-C score: ACLF-S (severe) for individuals with CLIF-C score ≥50 and ACLF-M (moderate) for individuals with CLIF-C scores <50.

All participants provided written informed consent. This study was conducted in accordance with the principles of the Declaration of Helsinki and was approved by the Ethics Committee of the Third Affiliated Hospital of Sun Yat-sen University (ethics approval number: RG2025-004-01).

### Preparation and characterization of MSC-Exos

Passage 4 BM-MSCs were cultured in T75 flasks until reaching 60%–70% confluence, and then, the medium was replaced with serum-free Dulbecco’s modified Eagle’s medium/Nutrient Mixture F-12 (DMEM/F12; Gibco, NY, USA), followed by cell culturing for another 24 h. The supernatant was processed using ultracentrifugation and size-exclusion chromatography (iZON, Christchurch, New Zealand) to isolate MSC-Exos (see Figure S1). These exosomes were resuspended in 100 μL of PBS (Biosharp, Hefei, China) for BCA assays (Beyotime, Shanghai, China) to determine protein concentrations and then stored at −80 °C for further use.

### Characterization of MSC-Exos

The purified MSC-Exos were characterized using nanoparticle tracking analysis (NTA), transmission electron microscopy (TEM), and western blotting (WB). For NTA, the particle size distribution and concentration were determined using a ZetaView instrument (Particle Metrix, Meerbusch, Germany). Samples were diluted in 1×  PBS and measured according to the manufacturer’s protocol. For TEM, the morphology was analyzed by adsorbing MSC-Exos onto Formvar/carbon-coated copper grids, followed by negative staining with 2% phosphotungstic acid (pH 6.8, Solarbio). Grids were imaged using a transmission electron microscope at 80 kV. The presence of canonical exosomal markers CD9, CD63, and Alix (all from Abcam, Cambridge, UK) and the absence of the negative marker GRP94 (Abcam) were confirmed by WB, as described in the WB section below.

### Animal model and treatment

Male BALB/c mice (4–6 weeks old, specific pathogen-free [SPF] grade) were purchased from Guangdong Medical Experimental Animal Center and housed under SPF conditions at the South China Agricultural University Animal Center. The ACLF model was established as described previously.^11^ Twelve mice were equally divided into the MSC-Exo and PBS control groups, with each animal receiving 100 µg of either exosomes or PBS (Biosharp) through the tail vein immediately after the final CCl_4_ injection. Twelve hours later, an abdominal injection of pentobarbital sodium (Sigma-Aldrich, MO, USA) at a dose of 50 mg/kg was performed for anesthesia. Subsequently, blood samples from the mouse eyeball and liver tissue were collected. Finally, the mice were euthanized using the cervical dislocation method. The study was approved by the Animal Ethics Committee of the South China Agricultural University (ethics approval number: no. 2024h052).

### Cell culture

Mouse macrophage cell lines RAW264.7 and J774.1 and normal mouse liver cell line AML12 were purchased from the American Type Culture Collection (Manassas, VA, USA). The BM-MSCs were obtained from the Human Center for Stem Cell Biology and Tissue Engineering, Key Laboratory for Stem Cells and Tissue Engineering (Guangzhou, Guangdong, China). The identification and characterization of MSCs were performed as previously described.^16^

AML12 cells were cultured in DMEM/F12 supplemented with 10% fetal bovine serum (FBS, Gibco), 1% insulin–transferrin–sodium selenite media supplement (Sigma-Aldrich), and 40 ng/mL dexamethasone (Sigma-Aldrich). RAW264.7 and J774.1 cells were cultured in DMEM/BASIC medium (Gibco) containing 10% FBS, 1% penicillin, and streptomycin (Solarbio, Beijing, China). Finally, BM-MSCs were cultured in an MSC-specific medium (StemCell Technologies, Vancouver, Canada). All cells were incubated at 37 °C with 5% CO_2_.

### Liver tissue staining

Briefly, 4–6 µm paraffin-embedded liver tissue sections were subjected to hematoxylin and eosin (H & E) staining, immunohistochemistry (IHC), immunofluorescence (IF), or TUNEL staining. Stained sections were imaged using a high-resolution pathology imaging system (TissueGnostics, Vienna, Austria). For IHC, sections were first de-paraffinized and subjected to antigen retrieval, followed by overnight incubation with primary antibodies against Mertk (Abcam) and Arid3a (Affinity, Shanghai, China) at 4 °C. Next, sections were incubated with HRP-conjugated secondary antibodies (Epizyme, Shanghai, China) at 23–25 °C for 1 h, and 3,3′-diaminobenzidine reagent (Epizyme) was used for visualization.

For IF, de-paraffinized sections underwent heat-induced epitope retrieval in EDTA buffer (Absin, Shanghai, China) using a microwave oven. Then, sections were incubated with primary antibodies against CD68 (Abcam), cleaved-caspase 3 (CST), CD86 (Abcam), CD206 (CST), and F4/80 (CST) overnight at 4 °C, followed by incubation with fluorescence-conjugated secondary antibodies (all from Abcam) at 23–25 °C for 1 h in the dark and then nuclear counterstaining with 4′,6-diamidino-2-phenylindole (DAPI; Beyotime). This analysis specifically investigated CD206, a classic marker of M2 macrophages, as well as CD86 and CD68, indicators of M1 macrophages.

TUNEL staining was performed using a TUNEL kit (ApeBio, Suzhou, China), with DAPI (Beyotime) used for nuclear staining.

### Efferocytosis index assessment

Macrophages from ACLF mouse liver tissue were labeled with CD68 (Abcam), and apoptotic cells were labeled with cleaved-caspase 3 (Abcam). Images were captured under a fluorescence microscope (ZEISS, Baden-Württemberg, Germany). The efferocytosis index for human tissue was calculated as the percentage of positive cells (CD68^+^ cells labeled with cleaved-caspase 3) relative to the total macrophage count in the field of view, as previously described.^18^

For mouse tissue, apoptotic cells were TUNEL-stained, and macrophages were labeled with F4/80 (CST). Images were captured under a fluorescence microscope (ZEISS). The efferocytosis index was the ratio of associated cells (apoptotic cells adjacent to or overlapping macrophages) to free cells (apoptotic cells distant from macrophages) within the field of view, as previously described.^19^

### Quantitative real-time polymerase chain reaction

Total RNA was extracted from cells or fresh liver tissue using an RNA/miRNA extraction kit (EZBioscience, CA, USA), and the concentration was determined using a NanoDrop spectrophotometer (Thermo Fisher Scientific, MA, USA). Next, the RNA was reverse-transcribed into cDNA with a reverse transcription kit (EZBioscience). To determine Cp values per sample, quantitative real-time polymerase chain reaction (qPCR) was performed using an LC480 system (Roche, Basel, Switzerland). *Gapdh* and *U6* were used as internal controls, and the 2^−ΔΔCT^ method was used for measuring relative expression. For specific primer sequences, see Table S3.

### Enzyme-linked immunosorbent assay

Serum alanine aminotransferase (ALT) and aspartate aminotransferase (AST) were assessed using enzyme-linked immunosorbent assay (ELISA) kits (BYabscience, Nanjing, China). Blood was collected and centrifuged at 2000× *g* for 10 min to isolate serum. Before testing, all reagents were equilibrated to room temperature (23–25 °C) for 30 min. Standards and samples were added to their respective wells, followed by 100 μL of HRP-conjugated antibody (Epizyme). The plate was sealed and incubated at 37 °C for 60 min and then washed five times with 300 μL wash buffer. Substrate solutions A and B (50 μL each) were added to each well and incubated at 37 °C for 15 min, followed by the addition of 50 μL stop solution. The absorbance was read at 450 nm using a microplate reader (TECAN, Männedorf, Switzerland), and AST and ALT concentrations were calculated based on standard curves.

### Western blotting

Proteins from tissues or cells were extracted using radioimmunoprecipitation assay buffer (RIPA; EpiZyme) supplemented with protease and phosphatase inhibitors (Beyotime). Protein concentrations were determined using a BCA assay kit (Thermo Fisher Scientific). After separation with sodium dodecyl sulfate polyacrylamide gel electrophoresis, samples were transferred onto polyvinylidene fluoride membranes (Millipore, MA, USA). Membranes were blocked with 5% milk for 1 h, followed by overnight incubation with primary antibodies (see Table S2) and then with secondary antibodies (Vazyme, Nanjing, China) for 1 h. Bands were visualized using a chemiluminescence system (Tanon, Shanghai, China).

### Induction of M1 macrophage polarization

Lipopolysaccharide (LPS)-induced polarization of macrophages toward the M1 phenotype is a classic model of inflammation. To induce the M1 inflammation model, RAW264.7 or J744.1 cells were seeded into six- or 12-well plates. Upon reaching approximately 60%–70% confluency, cells were stimulated for 12 h with LPS (Sigma-Aldrich) at a final concentration of 100 ng/mL.

### MSC-Exo co-culture experiment

M1 macrophages were incubated with MSC-Exos at 10 μg/mL per well for 24 h. The control group received an equivalent volume of PBS. The macrophage + MSC-Exo mixture was then treated with 1 μM Mertk-specific inhibitor UNC2541 (MCE, CA, USA) for 4 h.^20^

### In vitro phagocytosis assay

The assay was conducted using a phagocytosis kit (Cayman, MI, USA). After adding the Latex Beads-Rabbit IgG-FITC complex to the culture medium (1:400), the mixture was incubated at 37 °C and 5% CO_2_ for 2 h. Hoechst33342 (Beyotime) was used to stain nuclei for fluorescence microscopy (ZEISS). Phagocytic efficiency was assessed via flow cytometry; Phagocytic efficiency was defined as the percentage of macrophages that ingested fluorescent beads relative to total macrophage count. Data were acquired using a Cytoflex system (Beckman, CA, USA) and analyzed using FlowJo10.0 (Stanford University, CA, USA).

### In vitro efferocytosis assay

The assay followed published protocol.^21^ Normal AML12 cells were induced to undergo apoptosis using H_2_O_2_ (Sigma-Aldrich) and then stained with DAPI (Beyotime). Macrophages in 12-well plates were labeled with CMFDA (Abcam). Apoptotic cells and macrophages were mixed at a ratio of 5:1 and incubated at 37 °C in a 5% CO_2_ incubator for 2 h. After incubation, the medium was removed, and wells were washed thoroughly before imaging under a fluorescence microscope (ZEISS). Macrophages that engulfed more than one apoptotic cell were defined as positive cells. The efferocytosis index (proportion of positive cells to total macrophage count) was calculated using ImageJ (NIH, MD, USA).

### Dual-luciferase reporter assay

Plasmids for *Arid3a* overexpression (pCDNA3.1-*Arid3a*) and *Mertk* promoter (pGL3-Mertk) were constructed (see Supplementary material 4), then co-transfected with the pRL-TK plasmid into RAW264.7 cells using Lipo3000 (Thermo Fisher Scientific). After 24 h, cell lysates were collected for analysis. Luciferase activity was measured using a dual-luciferase reporter assay kit (Promega, WI, USA) and a microplate reader (TECAN). *Renilla* luciferase was used as the internal control.

### Transfection experiment to determine miRNA let-7a-5p function

Arid3a plays a crucial role in regulating gene expression and is downregulated by the let-7 miRNA family.^22^ Analysis by TargetScanHuman 8.0 (MIT, MA, USA) confirmed that let-7a-5p interacted with Arid3a at multiple sites to downregulate the transcription factor (https://www.targetscan.org/vert_80/). The function of let-7a-5p was then tested with transfection experiments.

RAW264.7 cells were seeded in six- or 12-well plates and treated with LPS to induce M1 polarization. Subsequently, let-7a-5p mimic (let-7a-5p sequence: TGAGGTAGTAGGTTGTATAGTT), let-7a-5p inhibitor, and their respective controls (RiboBio, Guangzhou, China) were transfected into cells using Lipo3000 (Thermo Fisher Scientific). At 24 h post-transfection, further experiments were performed. Primer sequences are shown in Table S3.

### Electroporation loading of miRNA

Using the Neon™ electroporation system (Thermo Fisher Scientific), 500 pmol of let-7a-5p mimic and its control (Riobio) were loaded into MSC-Exos, following published methods.^23^ Electroporation settings were 1000 V, 10 ms, and two pulses. Electroporated samples were incubated at 37 °C for 30 min to facilitate membrane recovery and then ultracentrifuged (10^5^ × *g* for 70 min at 4 °C) to remove excess unbound miRNA.

To calculate miRNA loading efficiency, a standard qPCR curve for let-7a-5p was established. The absolute quantity of let-7a-5p in MSC-Exo^let-7a-5p^ cells was determined. The following equation was used:


$$\begin{array}{l} \text{Loading efficiency }\\ =\left(\frac{{\text{let-7a-5p concentration in MSC-Exo}}^{\text{let-7a-5p}}}{\text{total amount of let-7a-5p}}\right)\times \text{ 100\%}. \end{array}$$

### Statistical analysis

All data were analyzed using GraphPad Prism 9.5 (San Diego, CA, USA). Data are presented as mean ± SEM of at least three replications. Between-group differences were determined with Student’s *t*-tests or one-way ANOVA. Relationships between variables were assessed using Spearman’s correlations. A correlation was considered strong at a correlation coefficient ≥0.8 (absolute value). Significance was set at *P *< .05.

## Results

### Impaired macrophage efferocytosis in ACLF liver tissues correlates with poor prognosis

**Figure 1.**
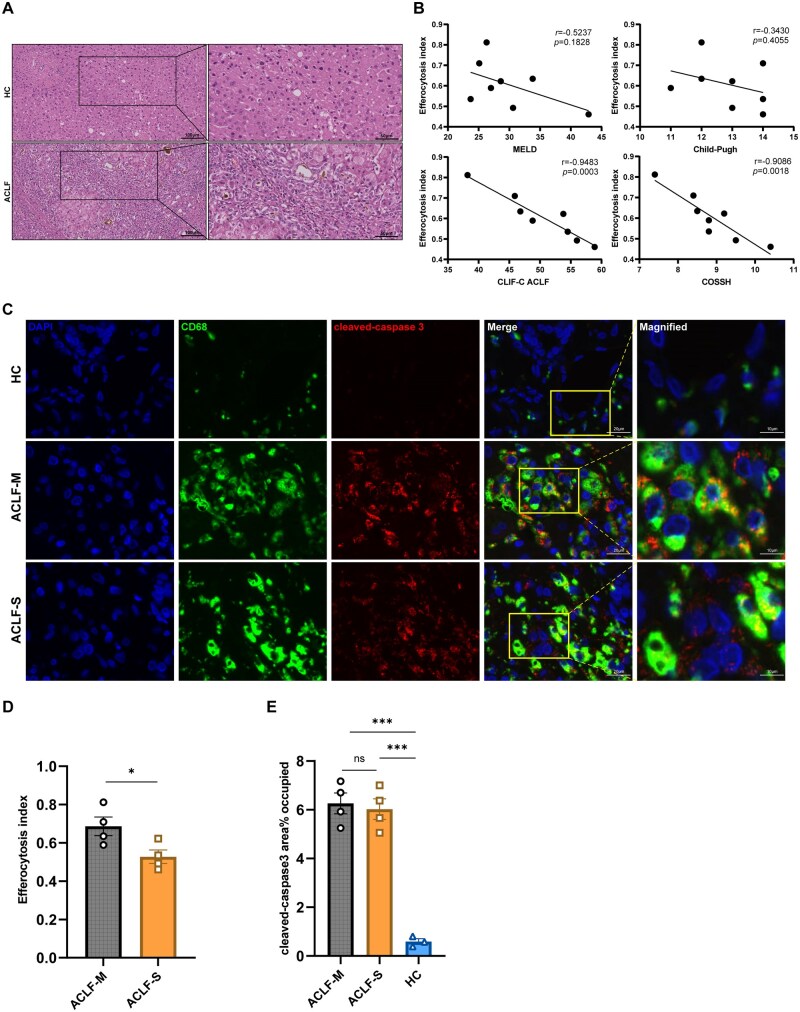
Impaired efferocytosis in liver tissues is linked to poor prognosis in patients with ACLF. **(**A) Representative H&E-stained images of liver tissue from patients with ACLF (*n* = 8) and healthy controls (*n* = 3). Magnification: 200×. Scale bar: 100 μm (main images); 50 μm (insets). (B) Spearman’s correlations between the efferocytosis index and relevant prognostic scores in patients with ACLF (*n* = 8). (C) Representative immunofluorescence staining images of liver tissue from patients with ACLF and healthy controls. ACLF-M (moderate) represents CLIF-C ACLF < 50 and ACLF-S (severe) represents CLIF-C ACLF ≥ 50. Macrophages were labeled with anti-CD68, apoptotic cells with anti-cleaved-caspase 3, and nuclei with DAPI.Magnification: 1000×. Scale bar: 20 μm (main images); 10 μm (insets). (D) Analysis of differences between the ACLF-M (*n* = 4) and ACLF-S (*n* = 4) groups in liver efferocytosis index (Student’s *t*-test). (E) Analysis of differences between patients and healthy controls (ACLF, *n* = 6; HC, *n* = 3) in percentage area of cleaved-caspase 3 (one-way ANOVA). Mean ± SEM, *P < .05, ***P < .001*. ns* indicates no significant difference.

H&E staining indicated that liver tissues of patients with ACLF displayed significantly more inflammation, necrosis, and fibrosis than HC liver tissue (Figure 1A). Additionally, we performed IF staining to determine the efferocytosis index in patients with ACLF. Results from correlation analyses revealed that efferocytosis negatively correlated with both CLIF-C and COSSH-ACLF scores. Hence, a lower macrophage efferocytosis in liver tissues was linked to a better prognosis in patients with ACLF (Figure 1B).

Next, we observed that the ACLF-S (severe) group had notably worse prognosis than the ACLF-M (moderate) group. These two groups also significantly differed in their efferocytosis index, suggesting that patients with worse clinical outcomes had a greater impairment of macrophage efferocytosis (Figure 1C and D). Furthermore, IF staining of liver tissues revealed a significant accumulation of apoptotic cells (cleaved-caspase 3^+^) and an increased presence of macrophages in both ACLF groups compared to those in the HC group (Figure 1C and E). Although the apoptotic burden was comparable between the ACLF-S and ACLF-M subgroups, the ACLF-S group exhibited a significantly lower efferocytosis index (Figure 1D), indicating a specific impairment in the clearance function of macrophages that correlates with disease severity.

### MSC-Exos alleviate inflammation and promote M2 macrophage polarization in the ACLF mouse model

The results of NTA on MSC-Exos revealed a concentration of 6.1 × 10^10^ particles/mL and a diameter between 40 and 160 nm, with a median of 142.4 nm. TEM indicated that these MSC-Exos were spherical, bilayered vesicles. WB analysis revealed that MSC-Exos expressed common exosomal surface markers, such as CD9, CD63, and ALIX, while GRP94 was not expressed^24^ (see Figure S1).

**Figure 2.**
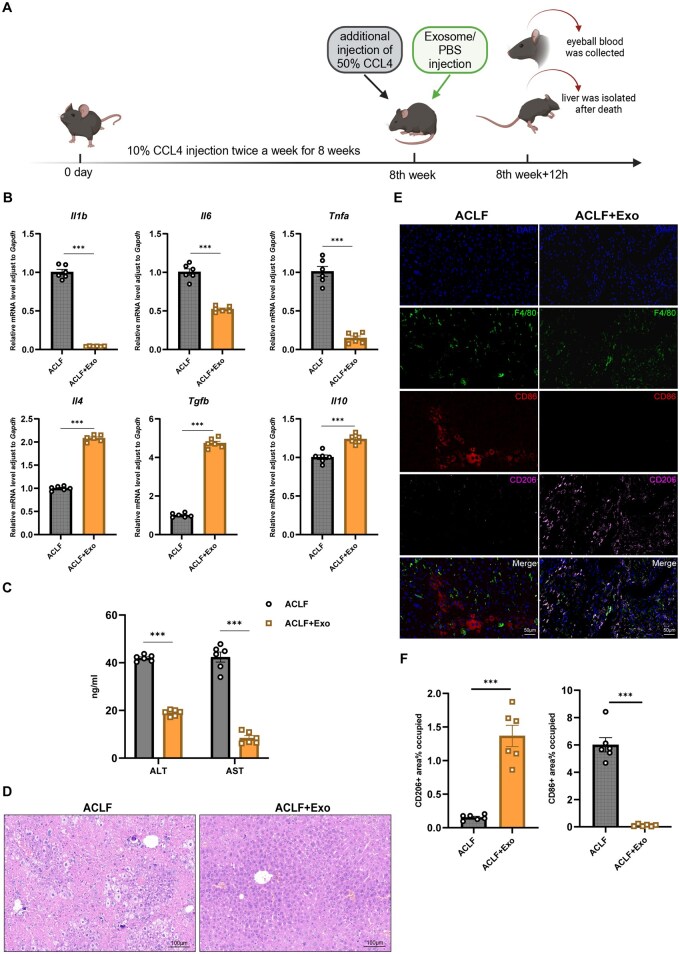
Mesenchymal stem cell–derived exosomes (MSC-Exos) alleviate inflammation and promote M2 polarization in the ACLF mouse model. (A) Timeline for establishing the ACLF mouse model and MSC-Exo treatment protocol. (B) qPCR of *Il1b, Il6, Tnfa, Il4, Il10, and Tgfb* mRNA expression in MSC-Exo and control groups (*n* = 6). (C) ELISA for serum ALT and AST levels in MSC-Exo and control groups (*n* = 6). (D) Representative H&E-stained images from MSC-Exo and control groups (*n* = 6). Magnification: 200×. Scale bar: 100 μm. (E) Representative immunofluorescence images from MSC-Exo and control groups (n = 6). Macrophages were labeled with anti-F4/80, M1 markers with anti-CD86, M2 markers with anti-CD206, and nuclei with DAPI. Magnification: 400×, Scale bar: 50 μm. (F) Differences between MSC-Exo and control mice in percentages of CD86- and CD206-positive areas (*n* = 6). Mean ± SEM, Student’s *t*-test; ***P < .001.

After treating ACLF model mice with MSC-Exos (Figure 2A), qPCR of liver samples revealed that the anti-inflammatory cytokines *Il4*, *Il10*, and *Tgfb* were upregulated in MSC-Exo mice, while pro-inflammatory cytokines *Il1b*, *Il6*, and *Tnfa* were downregulated (Figure 2B). Next, ELISA showed that serum AST and ALT levels were significantly reduced in the MSC-Exo group (Figure 2C). Subsequent H & E staining of liver tissues demonstrated that this group also exhibited a remarkable decrease in inflammation and necrosis compared with those in control mice (Figure 2D).

IF staining of liver tissues demonstrated that MSC-Exo mice expressed significantly more CD206 and less CD86 than control mice (Figure 2E and F), suggesting a shift toward M2 polarization in liver macrophages after MSC-Exo infusion.

In summary, these findings indicated that MSC-Exo administration in the ACLF mouse model significantly mitigated liver inflammation, reduced serum transaminase levels, and promoted M2 polarization of macrophages.

### MSC-Exos enhance macrophage efferocytosis in liver tissues of ACLF mouse model

**Figure 3.**
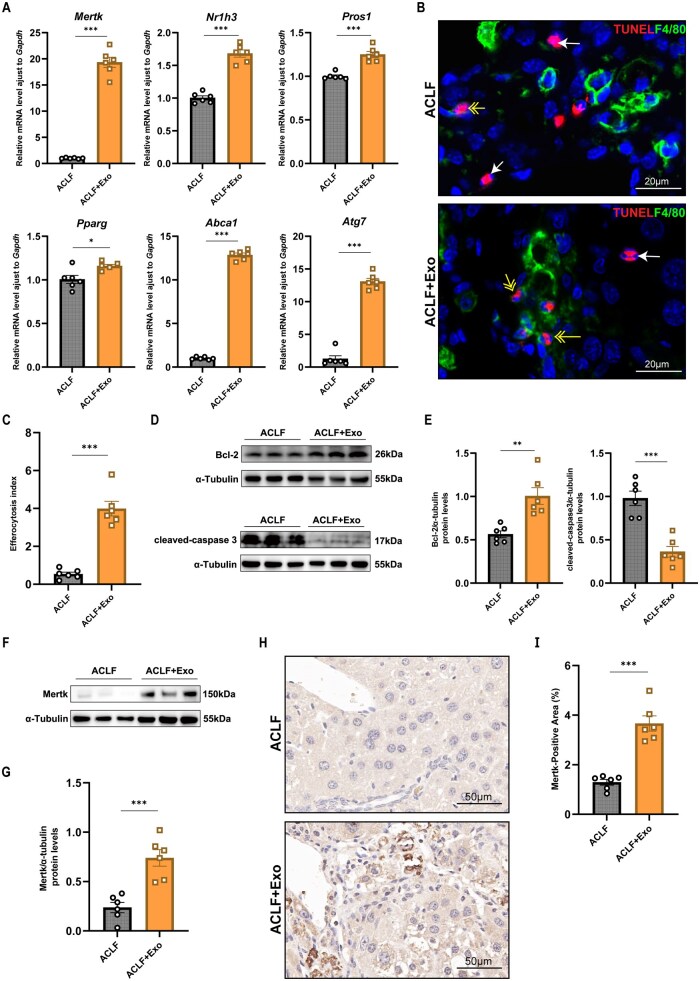
MSC-Exos enhance macrophage efferocytosis in liver tissues of ACLF mouse model. (A) mRNA levels of efferocytosis-related genes in liver tissues from MSC-Exo and control groups were quantified by qPCR (*n* = 6). (B) Representative IF staining images of liver tissues from MSC-Exo and control groups (n = 6). Macrophages were labeled with anti-F4/80, apoptotic cells were detected by TUNEL assay, and nuclei were counterstained with DAPI. Double-headed arrows indicate associated apoptotic cells (adjacent to or overlapping with macrophages); single-headed arrows indicate free apoptotic cells (distant from macrophages). The efferocytosis index was calculated as the ratio of associated to free cells per field of view. Magnification: 1000×. Scale bar: 20 μm. (C) Quantitative analysis of the efferocytosis index in liver tissues from MSC-Exo and control groups (*n* = 6). (D, E) Western blotting of Bcl-2 and cleaved-caspase 3 expression in liver tissues from MSC-Exo and control groups, normalized to α-tubulin. Relative band intensities were quantified in ImageJ (*n* = 6). (F, G) Western blotting of Mertk expression in liver tissues from MSC-Exo and control groups, normalized to α-tubulin. Relative band intensities were quantified in ImageJ (*n* = 6). (H, I) IHC staining of Mertk expression in liver tissues from MSC-Exo and control groups, quantified in ImageJ (*n* = 6). Magnification: 400×. Scale bar: 50 μm. Mean ± SEM, Student’s *t*-test; *P < .05, **P < .01, ***P < .001.

We next asked whether MSC-Exos could modulate macrophage polarization in an LPS-induced M1 inflammatory model.^25^ The results of qPCR demonstrated an overall upregulation of efferocytosis-related genes in MSC-Exo livers compared with that in control livers. Notably, *Mertk* expression exhibited the most significant increase (Figure 3A).

Subsequent IF staining to assess the efferocytosis index in liver tissues demonstrated that the MSC-Exo group had a significantly higher index than the controls (Figure 3B and C). Additionally, WB indicated that Bcl-2 concentration increased in the MSC-Exo group, while cleaved-caspase 3 concentration decreased (Figure 3D and E). WB and IHC analyses also confirmed the qPCR findings of significant Mertk upregulation in the MSC-Exo group compared with that in the control group (Figure 3F–I).

Collectively, these findings demonstrated that MSC-Exos enhanced macrophage efferocytosis in the liver tissues of ACLF mice, accompanied by increased Mertk expression.

### MSC-Exos promote M2 polarization, enhance efferocytosis, and upregulate Mertk in vitro

**Figure 4.**
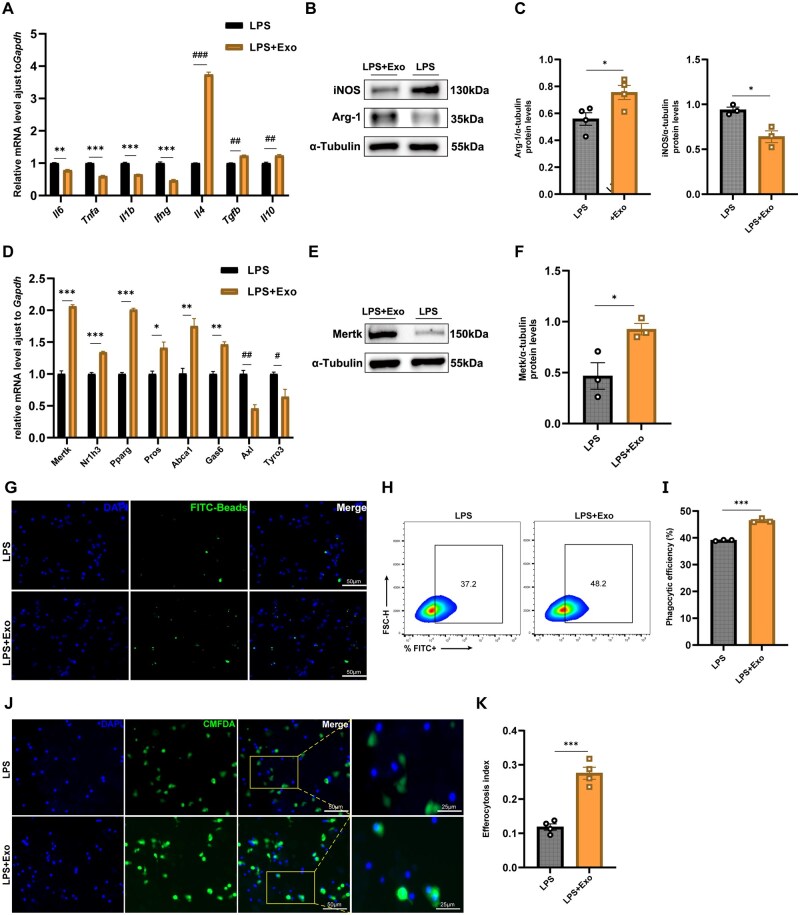
MSC-Exos promote M2 polarization and enhance efferocytosis via upregulating Mertk in macrophages in vitro. (A) qPCR of *Il6, Tnfa, Il1b, Ifng, Il4, Tgfb,* and *Il10* mRNA expression in MSC-Exo and control groups (*n* = 3). (B, C) Western blotting of Arg-1 and iNOS expression in RAW264.7 cells from MSC-Exo and control groups, normalized to α-tubulin. Relative band intensities were quantified in ImageJ (*n* = 3 or 4). Arg-1: M2 macrophage marker. iNOS: M1 macrophage marker. (D) mRNA expression of efferocytosis-related genes in RAW264.7 cells from MSC-Exo and control groups, quantified by qPCR (*n* = 3). (E, F) Western blotting of Mertk expression in RAW264.7 cells from MSC-Exo and control groups, normalized to α-tubulin. Relative band intensities were quantified in ImageJ (*n* = 3). (G) Representative fluorescence microscopy images of RAW264.7 cells from MSC-Exo and control groups in the phagocytosis assay (*n* = 3). Magnification: 200×. Scale bar: 50 μm. (H, I) Phagocytosis assay in LPS-stimulated RAW264.7 macrophages ± MSC-Exo pretreatment. Cells gated on FSC-A/SSC-A for macrophages; FITC^+^ (FITC-positive) events indicate bead internalization after a 2-h incubation. Mean ± SEM (*n* = 3). (J) Representative fluorescence microscopy images of in vitro efferocytosis assays. Magnification: 200×. Scale bar: 50 μm (main images); 25 μm (insets). (K) Quantitative analysis of the efferocytosis index in RAW264.7 cells from MSC-Exo and control groups (*n* = 4). Mean ± SEM, Student’s *t*-test; *P < .05, **P < .01, ***P < .001, *^#^P* < .05, *^##^P <* .01, *^###^P <* .001.

Results of qPCR for our M1 polarization/inflammation cell model indicated that anti-inflammatory cytokines *Il4*, *Il10*, and *Tgfb* were upregulated in the MSC-Exo group, while pro-inflammatory cytokines *Il6*, *Tnfa*, *Il1b*, and *Ifng* were downregulated (Figure 4A). Additionally, WB demonstrated that the M2 marker Arg-1 was upregulated, while the M1 marker iNOS was downregulated in the MSC-Exo group (Figure 4B and C).

The mRNA expression of key efferocytosis-related genes, including *Mertk*, was also elevated in the MSC-Exo group (Figure 4D). WB results confirmed the significant increase in Mertk expression in RAW264.7 cells of the MSC-Exo group (Figure 4E and F).

Our phagocytosis assay revealed that RAW264.7 cells of MSC-Exo engulfed more beads than control macrophages, as assessed using fluorescence microscopy and flow cytometry (Figure 4G–I). Subsequently, we successfully induced apoptosis in AML12 cells in vitro by stimulating them with H_2_O_2_ (see Figure S2). After co-culturing apoptotic AML12 cells with either MSC-Exo or control macrophages, we observed a higher efferocytosis index in the MSC-Exo group (Figure 4J and K).

Taken together, our data demonstrated that MSC-Exos promoted M2 polarization in the M1-polarized RAW264.7 cells, enhanced efferocytosis, and increased Mertk expression.

### MSC-Exos enhance macrophage efferocytosis primarily through upregulating Mertk

After co-culture with M1-polarized RAW264.7 cells, MSC-Exos increased *Mertk* mRNA while downregulating the expression of *Axl and Tyro3*, the other two TAM receptor members (Figure 4D).

**Figure 5.**
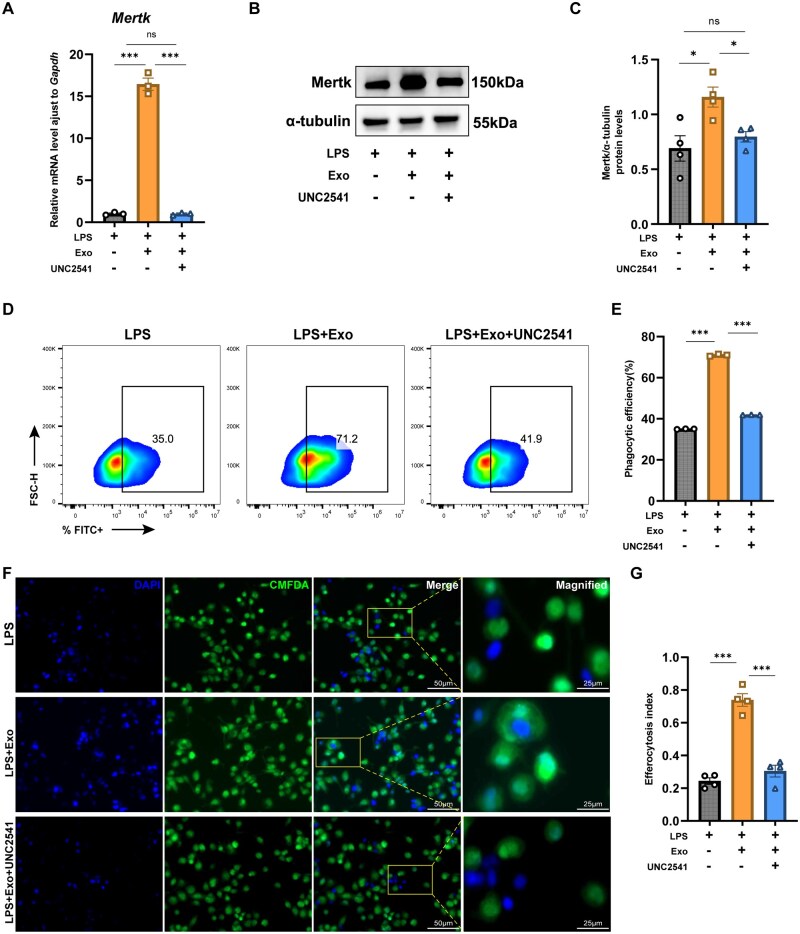
MSC-Exos enhance macrophage efferocytosis primarily through upregulation of Mertk expression. (A) qPCR of *Mertk* mRNA expression in J774.1 cells from different groups, with or without UNC2541 (*n* = 3). (B, C) Western blotting of Mertk expression in J774.1 cells from different groups, with or without UNC2541, normalized to α-tubulin. Relative band intensities were quantified in ImageJ (*n* = 4). (D, E) Flow cytometry of phagocytic efficiency after in vitro phagocytosis assays with or without UNC2541 in J774.1 cells from different groups (*n* = 3). (F) Representative fluorescence microscopy images of in vitro efferocytosis assays. Magnification: 200×. Scale bar: 50 μm (main images); 25 μm (insets). (G) Quantitative analysis of the efferocytosis index in J774.1 cells from different groups with or without UNC2541 (*n* = 4). Mean ± SEM, one-way ANOVA; *P < .05, ***P < .001, *ns* indicates no significant difference.

To verify whether MSC-Exo-enhanced efferocytosis in M1 macrophages was Mertk-dependent, we induced M1 polarization in J774.1 cells before co-culturing them with MSC-Exos. After treatment with Mertk-specific inhibitor UNC2541, qPCR and WB analyses revealed a significant decrease in Mertk expression (Figure 5A–C).

Subsequent phagocytosis assays and flow cytometry demonstrated that treatment with UNC2541 significantly suppressed phagocytic efficiency in J774.1 cells of the MSC-Exo group (Figure 5D and E). Furthermore, UNC2541 treatment significantly inhibited the efferocytosis index in J774.1 cells of the MSC-Exo group (Figure 5F and G). These findings suggested that MSC-Exo enhancement of efferocytosis was primarily dependent on the upregulation of Mertk expression.

### Transcription factor Arid3a negatively regulates Mertk expression, and let-7a-5p enhances macrophage efferocytosis via the Arid3a/Mertk axis

**Figure 6.**
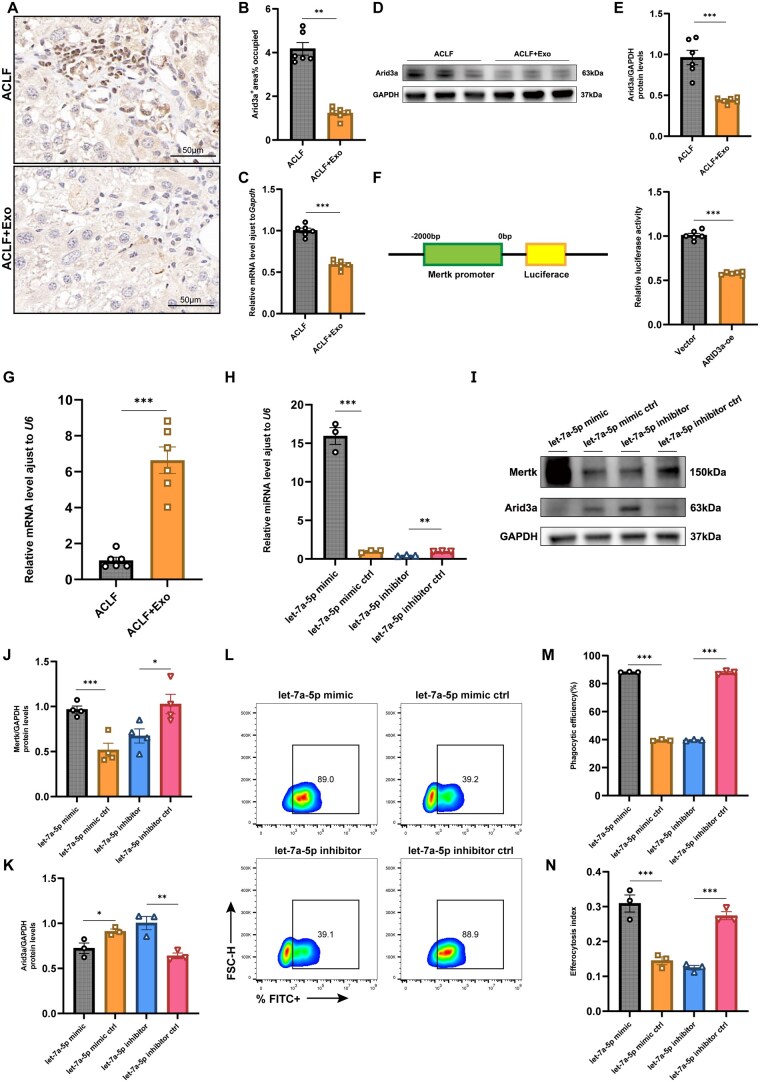
MicroRNA let-7a-5p enhances macrophage efferocytosis via downregulation of Arid3a, a Mertk inhibitor. (A, B) IHC staining of Arid3a expression in liver tissues from MSC-Exo and control groups, quantified by ImageJ (*n* = 6). Magnification: 400×. Scale bar: 50 μm. (C) qPCR of *Arid3a* mRNA expression in liver tissues from MSC-Exo and control groups (*n* = 6). (D, E) Western blotting of Arid3a expression in  liver tissues from MSC-Exo and control groups, normalized to GAPDH. Relative band intensities were quantified by ImageJ (*n* = 6). (F) Dual-luciferase reporter assay in RAW264.7 cells co-transfected with *Mertk* promoter plasmid and *Arid3a* overexpression plasmid or pGL3 empty control plasmid (*n* = 6). (G) qPCR analysis of let-7a-5p expression in liver tissues from MSC-Exo and control groups in the ACLF mouse model (*n* = 6). (H) qPCR validation of let-7a-5p expression levels in M1-polarized RAW264.7 cells after transfection with let-7a-5p mimic, inhibitor, or their respective controls (*n* = 3). (I–K) Western blotting of Arid3a and Mertk expression in RAW264.7 cells from different treatment groups, normalized to GAPDH. Relative band intensities were quantified by ImageJ (*n* = 4). (L, M) Flow cytometry analysis of phagocytic efficiency in RAW264.7 cells from different groups after in vitro phagocytosis assays (*n* = 3). (N) Quantitative analysis of the efferocytosis index in RAW264.7 cells from different groups after in vitro efferocytosis assays (*n* = 3). Mean ± SEM. Statistical significance was determined by Student’s *t*-test or one-way ANOVA; *P < .05, **P < .01, ***P < .001, ns indicates no significant difference.

In our in vivo experiments, the MSC-Exo group had significantly lower Arid3a expression than the control group, according to the results of IHC, qPCR, and WB (Figure 6A–E). Our in vitro experiments supported the in vivo findings; dual-luciferase reporter assays showed that Arid3a binds to the promoter region of Mertk and negatively regulates its expression (Figure 6F).

Subsequently, we examined the expression of let-7a-5p in liver tissues from mice in our in vivo experiments. qPCR analysis revealed a significantly higher expression of let-7a-5p in the MSC-Exo group than in the control group (Figure 6G). After determining that let-7-5p downregulated Arid3a expression via interactions at multiple sites, we transiently transfected M1-polarized RAW264.7 cells with let-7a-5p mimic, let-7a-5p inhibitor, or their controls. Successful transfection of let-7a-5p into macrophages was confirmed via qPCR (Figure 6H). WB demonstrated that Arid3a expression was downregulated and Mertk expression was upregulated in the let-7a-5p mimic group, whereas the opposite was observed in the let-7a-5p inhibitor group (Figure 6I–K).

Phagocytosis assays indicated that the let-7a-5p mimic group had higher phagocytic efficiency than the control, whereas the inhibitor group had lower efficiency (Figure 6L and M). Additionally, the efferocytosis index was significantly higher in the let-7a-5p mimic group than in the control and significantly lower in the inhibitor group (Figure 6N). These results indicate that let-7a-5p suppresses Arid3a expression, thus upregulating Mertk expression and enhancing macrophage efferocytosis.

### Engineered MSC-Exos^let-7a-5p^ enhance macrophage efferocytosis more effectively than unloaded MSC-Exos

**Figure 7.**
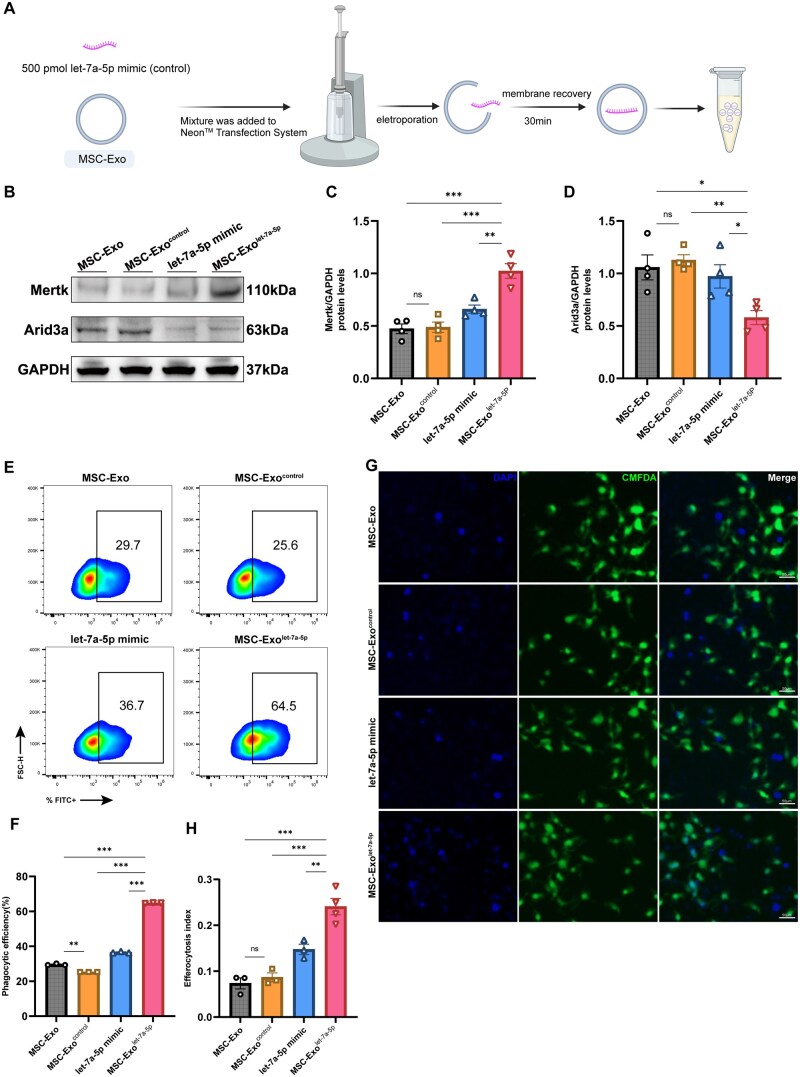
Engineered MSC-Exos^let-7a-5p^ enhance macrophage efferocytosis more effectively than unloaded MSC-Exos in vitro. (A) Schematic of electroporation procedure for loading let-7a-5p into MSC-Exos. (B–D) M1-polarized RAW264.7 cells were co-cultured with unloaded MSC-Exos, MSC-Exos^let-7a-5p^, MSC-Exos^control^, or an equivalent amount of let-7a-5p. Arid3a and Mertk expression was assessed by western blotting and normalized to GAPDH expression. Relative band intensities were quantified in ImageJ (*n* = 3). (E, F) Phagocytic efficiency in RAW264.7 cells from different groups was evaluated using flow cytometry after in vitro phagocytosis assays (*n* = 3). (G, H) Fluorescence microscopy and quantitative analysis of the efferocytosis index in RAW264.7 cells from different groups after efferocytosis assays (*n* = 3). Mean ± SEM, one-way ANOVA; *P < .05, **P < .01, ***P < .001, *ns* indicates no significant difference.

Standard curve analysis revealed a ∼13.4% loading efficiency of let-7a-5p mimics into MSC-Exos (see Figure 7A and Figure S2). To compare the efferocytosis capacity of M1 macrophages co-cultured with MSC-Exos^let-7a-5p^, MSC-Exos^control^, unloaded MSC-Exos, and an equivalent amount of let-7a-5p mimics as contained in MSC-Exos^let-7a-5p^, we conducted subsequent experiments.

After co-culturing MSC-Exos^let-7a-5p^ with M1-polarized RAW264.7 cells, WB revealed that Mertk expression was significantly more upregulated in these macrophages than in the MSC-Exos^control^, unloaded MSC-Exos, and let-7a-5p mimic groups, whereas Arid3a expression was reduced (Figure 7B–D). Phagocytosis assays and flow cytometry of these groups indicated that RAW264.7 cells from the MSC-Exos^let-7a-5p^ group exhibited superior phagocytic efficiency (Figure 7E and F). Furthermore, these RAW264.7 cells also had a higher efferocytosis index than the other three groups (Figure 7G and H). Collectively, these findings demonstrate that engineered MSC-Exos^let-7a-5p^ exhibit markedly enhanced macrophage efferocytosis compared to unloaded MSC-Exos, predominantly through the Arid3a/Mertk axis.

## Discussion

Attenuated efferocytosis is observed in various liver diseases, such as drug-induced liver injury,^26^ liver ischemia-reperfusion injury,^27^ and cholestatic liver disease.^28^ In our study, we found that ACLF liver tissue showed a significant increase in apoptotic cell accumulation and macrophage count compared with those in normal liver tissue. This expansion of the hepatic macrophage pool involves both proliferation of resident KCs and recruitment of blood-derived monocytes, which differentiate into macrophages upon entering the liver. However, in ACLF, this increase in macrophages does not lead to the removal of apoptotic cells, suggesting that impaired efferocytosis is the cause of symptoms, such as inflammation and secondary necrosis. The ACLF-S and ACLF-M groups had a similar apoptotic burden. However, the significantly impaired efferocytosis in ACLF-S strongly suggests that the worse prognosis in severe ACLF is driven not by a higher rate of apoptosis, but by a fundamental defect in the clearance machinery itself. This functional deficit leads to the accumulation of uncleared apoptotic cells, which subsequently undergo secondary necrosis, triggering intense inflammation and organ damage—the hallmark of ACLF progression.^29^ Our study revealed a strong correlation between efferocytosis impairment and two prognostic models (CLIF-C ACLF and COSSH-ACLF). These findings highlight the reduced efficiency of macrophage infiltration in ACLF and its association with adverse clinical outcomes.

MSCs and their derived exosomes enhance macrophage efferocytosis in various diseases.^30–32^ In our study, administering MSC-Exos to ACLF mice notably improved liver pathology and reduced transaminase levels. Treatment with MSC-Exos downregulated CD86 expression and upregulated CD206 expression, which reflects a shift from the M1 to M2 macrophage phenotype. Previous studies on ACLF have shown that MSCs promote M2 polarization via upregulating Mertk expression. Mertk plays a critical role in efferocytosis,^33^ and the underlying mechanism involves anti-inflammatory action and macrophage polarization, specifically suppressing NF-κB signaling and thus inhibiting M1 polarization.^34^ Mertk also facilitates reparative M2 polarization,^35–38^ which enhances dead cell removal because the M2 phenotype has greater efferocytotic capacity than the M1 phenotype.^39^^,^^40^ Our investigation identified significant upregulation of efferocytosis-related genes, especially *Mertk*, in the liver tissues of the MSC-Exo group. We also observed an upregulation of the anti-apoptotic marker Bcl-2 and downregulation of the apoptotic marker cleaved-caspase 3. Together, these findings indicated that MSC-Exos enhanced macrophage efferocytosis in vivo.

We conducted in vitro experiments to investigate the effects of MSC-Exos on macrophage efferocytosis. Co-culturing MSC-Exos with M1 macrophages significantly upregulated the M2 marker Arg-1 and downregulated the M1 marker iNOS. This shift was accompanied by a decrease in pro-inflammatory cytokines and an increase in anti-inflammatory cytokines. Overall, our findings highlighted the role of MSC-Exos in promoting M2 macrophage polarization.

Mertk belongs to the TAM receptor family, which includes Axl and Tyro3. Treatment with MSC-Exos upregulated Mertk and other efferocytosis-related genes, leading to greater efferocytotic efficiency, whereas Axl and Tyro3 expression showed a downward trend. This suggests that MSC-Exos promote M2 polarization and enhance efferocytosis primarily through specific upregulation of Mertk. Existing reports on Axl expression in polarized macrophages appear conflicting. One study observed increased Axl mRNA in dexamethasone-treated human monocyte-derived M2 macrophages,^41^ whereas another detected upregulated Axl expression in LPS-induced M1-polarized bone marrow-derived macrophages.^42^ Studies on Tyro3 are relatively limited, with some indicating higher expression in M2 macrophages,^43^ though we observed a decreasing trend in our M2-favorable conditions. These discrepancies could be attributed to differences in cellular origin or stimulation protocols. Furthermore, after treatment with the Mertk-specific inhibitor UNC2541, macrophage efferocytosis was significantly inhibited. This finding indicates that MSC-Exo-mediated enhancement of macrophage efferocytosis is Mertk-dependent. Taken together, because managing ACLF requires prompt intervention to lower inflammation,^44^ our results suggest that MSC-Exos may be a valuable tool for improving therapeutic strategies.

The transcription factor Arid3a inhibits Mertk expression to minimize macrophage efferocytosis in cholestatic liver disease.^14^ In our in vivo animal experiments, we found that MSC-Exo treatment significantly downregulated Arid3a mRNA and protein in liver tissue, and we used dual-luciferase reporter assays to confirm that Arid3a negatively regulates Mertk expression and is itself downregulated by let-7 family miRNAs, specifically let-7a-5p.^15^ As a natural component of MSC-Exos,^16^ let-7a-5p downregulation in ACLF is associated with patient mortality within 30 days.^45^ Additionally, treatment with let-7a-5p in acute lung injury alleviated pulmonary inflammation.^46^ Our in vitro experiments showed that transfecting let-7a-5p mimic into M1 macrophages led to Arid3a downregulation and Mertk upregulation, along with a notable increase in phagocytic efficiency and the efferocytosis index. These findings confirm the importance of let-7a-5p as an efferocytosis promoter.

Engineered exosomes loaded with drugs or oligonucleotides are exciting therapeutic tools that have been applied to various diseases.^47–49^ Exosomes derived from MSCs inherently contain a wealth of active components with anti-inflammatory and regenerative capabilities, making them attractive drug delivery vehicles.^50^^,^^51^ In our investigation, we loaded let-7a-5p and its control into MSC-Exos using electroporation. To test effectiveness as drug carriers, they were compared with unloaded MSC-Exos and an equivalent amount of let-7a-5p mimic in terms of their effects on M1 macrophages. Despite a modest let-7a-5p loading rate of 13.4%, the MSC-Exos^let-7a-5p^ group significantly inhibited Arid3a expression and increased Mertk expression, while also enhancing phagocytic efficiency and efferocytosis index.

This study has several limitations, including the low efficiency of loading miRNA, which may require the optimization of related experimental conditions in future research. Additionally, the MSC-Exo^let-7a-5p^ has not yet been validated through in vivo experiments, an issue that will be addressed in subsequent studies.

## Conclusion

Our findings demonstrate that let-7a-5p serves as a promising therapeutic target for ACLF by enhancing efferocytosis through the Arid3a/Mertk axis. Positive outcomes from these experiments would validate the therapeutic potential of MSC-Exos^let-7a-5p^, as well as provide a robust foundation for clinical translation, offering a novel nanomedicine-based strategy for ACLF treatment. Future studies should prioritize optimizing the loading efficiency of engineered MSC-Exos and validating their therapeutic efficacy in in vivo ACLF models.

## Supplementary Material

szaf058_Supplementary_Data

## Data Availability

All data are available from the corresponding authors upon reasonable request.
