# Supplementary material for: Mesenchymal stem cell–derived exosome delivery of let-7a-5p enhances macrophage efferocytosis via Arid3a/Mertk axis in acute-on-chronic liver failure
Source: Stem Cells Transl Med. 2025 Dec 2;14(12):szaf058. doi: 10.1093/stcltm/szaf058 (PMC12673206; doi:10.1093/stcltm/szaf058)

**Supplementary Information**

**Mesenchymal stem cell-derived exosome delivery of let-7a-5p enhances macrophage efferocytosis via Arid3a/Mertk axis in acute-on-chronic liver failure**

Junyi Wang, M.D.^1,2#^, Zhihui Li, Ph.D.^1,2#^, Zhouhan Wang, M.D.^1,2#^, Wei Liang, M.D.^1,2#^, Shibo Meng, M.S.^1,2^, Junfeng Chen, M.D.^1,2^, Jialei Wang, M.S.^1,2^, Jing Zhang, Ph.D.^1,2*^ and Bingliang Lin, M.D., Ph.D.^1,2,3*^

^1^Department of Infectious Diseases, The Third Affiliated Hospital of Sun Yat-sen University, Guangzhou, Guangdong 510630, China

^2^Guangdong Key Laboratory of Liver Disease Research, The Third Affiliated Hospital of Sun Yat-sen University, Guangzhou 510630, China

^3^Key Laboratory of Tropical Disease Control (Sun Yat-sen University), Ministry of Education, Guangzhou, Guangdong 510080, China

^#^These authors contributed equally to this work.

^*^Corresponding authors:

Jing Zhang, Department of Infectious Diseases, The Third Affiliated Hospital of Sun Yat-sen University, No.600 Tianhe Road, Guangzhou, 510630, Guangdong, China; Telephone: +8620-85253165, E-mail: zhangj75@mail2.sysu.edu.cn

Bingliang Lin, Department of Infectious Diseases, The Third Affiliated Hospital of Sun Yat-sen University, No.600 Tianhe Road, Guangzhou, 510630, Guangdong, China; Telephone: +8620-85253165, Fax: +8620-85252259, E-mail: linbingl@mail.sysu.edu.cn

| **Table S1. Clinical Characteristics of the ACLF and HC Groups** | | | |
| --- | --- | --- | --- |
| **Clinical Characteristics** | **ACLF**  **(*n* = 8)** | **HC**  **(*n* = 3)** | ***p* value** |
| Age (years) | 41.13 ± 3.29 | 43.33 ± 1.76 | 0.7047 |
| Gender (Female/ Male) | 0/8 | 0/3 | / |
| TBil (μmol/L) | 484.40 ± 23.86 | 15.90 ± 7.51 | <0.001 |
| DBil (μmol/L) | 275.40 ± 17.98 | 3.14 ± 1.00 | <0.001 |
| ALT (U/L) | 50.00 ± 6.83 | 23.00 ± 7.21 | 0.0541 |
| AST (U/L) | 103.60 ± 14.98 | 19.00 ± 3.79 | 0.0088 |
| PT (s) | 38.15 ± 5.37 | 13.17 ± 0.35 | 0.0121 |
| INR | 4.07 ± 0.79 | 1.00 ± 0.02 | 0.0121 |
| Cr (μmol/L) | 73.00 ± 13.77 | 70.67 ± 6.33 | 0.9232 |
| ALB (g/L) | 34.98 ± 1.61 | 45.93 ± 0.82 | 0.0032 |
| WBC (×10^9^/L) | 7.16 ± 1.03 | 5.67 ± 0.08 | 0.4164 |
| HE-grade (1/2/3/4) | 0/3/1/4 | N/A | / |
| Ascites-grade (0/1/2/3) | 1/1/0/6 | N/A | / |

**HE**: hepatic encephalopathy. **Ascites-grade**: 0 represents no ascites, 1 represents a small amount of ascites, 2 represents a moderate amount of ascites, and 3 represents a large amount of ascites. **N/A**: not applicable. Data are shown as mean ± SEM. Statistical analysis: Normality was assessed using Shapiro–Wilk test. Non-normally distributed continuous variables were analyzed using Mann–Whitney U test. Normally distributed variables were compared using Student’s *t*-test (equal variances) or Welch’s *t*-test (unequal variances), with variance homogeneity determined by F test. Data are expressed as mean ± SEM.

**Table S2. Primary antibodies of WB/IHC/IF**

| Name | Resource |
| --- | --- |
| CD68 | ab213363 (Abcam) |
| Cleaved-caspase 3 | 9664 (CST) |
| F4/80 | 30325 (CST) |
| CD86 | Ab119857 (Abcam) |
| CD206 | 24595 (CST) |
| CD9 | Ab307085 (Abcam) |
| CD63 | Ab315108 (Abcam) |
| Alix | Ab275377 (Abcam) |
| GRP94 | Ab238126 (Abcam) |
| Bcl-2 | 3498 (CST) |
| Mertk | Ab184086 (Abcam) 150kDa, # DF7344 (Affinity) 110kDa |
| iNOS | Ab178945 (Abcam) |
| Arg-1 | Ab233548 (Abcam) |
| Arid3a | DF12558 (Affinity) |
| α-tubulin | 2144 (CST) |
| GAPDH | LF205 (Epizyme) |
| Goat anti-rabbit IgG (FITC) | Ab6717 (Abcam) |
| Goat anti-rabbit IgG (Alexa Fluor 647) | Ab150079 (Abcam) |
| Goat anti-rabbit IgG (Cy3) | Ab6939 (Abcam) |

**Table S3. Primers used for qPCR**

| Gene Name | Species | Forward Sequences | Reverse Sequences |
| --- | --- | --- | --- |
| *Il1b* | Mouse | CACTACAGGCTCCGAGATGAACAAC | TGTCGTTGCTTGGTTCTCCTTGTAC |
| *Il6* | Mouse | CTCCCAACAGACCTGTCTATAC | CTCCCAACAGACCTGTCTATAC |
| *Tnfa* | Mouse | CACGCTCTTCTGTCTACTGAACTTC | CTTGGTGGTTTGTGAGTGTGAGG |
| *Il4* | Mouse | TACCAGGAGCCATATCCACGGATG | TGTGGTGTTCTTCGTTGCTGTGAG |
| *Il10* | Mouse | GGACAACATACTGCTAACCGACTC | ATTTCCGATAAGGCTTGGCAACC |
| *Tgfb* | Mouse | ACCGCAACAACGCCATCTATGAG | GGCACTGCTTCCCGAATGTCTG |
| *Mertk* | Mouse | CCTAACCGTACCTGGTCTGAC | GGGAGGGGATTACTTTGATGTTG |
| *Abca1* | Mouse | GAGGAGCAGGGCATTGGAGTG | ACAGCATCATGGAGACCGAAGTG |
| *Atg7* | Mouse | TCTGGGAAGCCATAAAGTCAGG | GCGAAGGTCAGGAGCAGAA |
| *Nr1h3* | Mouse | TCCGCCGCAGTGTCATCAAG | CCGCCGCATGTAGGTGTCC |
| *Pparg* | Mouse | AGCCCTTCACTACTGTTGACTTCTC | CTGCAACCACTGGATCTGTTCTTG |
| *Pros1* | Mouse | CTGAGGGTTGGCATGTAAATGTGAC | GCAAAGGGCACTGTGTTGTTACC |
| *Ifng* | Mouse | CTGGAGGAACTGGCAAAAGGATG | CAGGTGTGATTCAATGACGCTTATG |
| *Gas6* | Mouse | CCGCGCCTACCAAGTCTTC | CGGGGTCGTTCTCGAACAC |
| *Axl* | Mouse | GGACCACTGAAGCCACCTTGAAC | GCCACCTTATGCCGATCTACCATG |
| *Arid3a* | Mouse | CTCAACCTGCCTACCTCCATCAC | GCCTCGCCTCTCACACTCATAG |
| *Gapdh* | Mouse | AGGTCGGTGTGAACGGATTTG | TGTAGACCATGTAGTTGAGGTCA |
| *let-7a-5p* | Mouse | CCGCTGAGGTAGTAGGTTGTATAGTT | |
| *U6* | Mouse | CTCGCTTCGGCAGCACA | AACGCTTCACGAATTTGCGT |

**Supplementary Material 4. The Promoter Sequence of Mertk**

ggtaccgagctctcccgctggggtcactccaggtgctagatactcaaggtcacagacagagaggccacagagaaaagacaatccatttcctctgtccaaatctatgaatctaataaaatggccagtgcttcattcctactgcattggtagtagcctataa

aacagtactgtacaagggaggccccattaacaaagacagatttgcggctggaaagttggctcagtgcttaagagcacttg

ttgctcttgcagaggaccagtgttaaattcacagtacccaactggtagctcacaagtatctgcaactacattctaggaga

cccaacaccctcttcagacctccatgggtaccccagcatacacatgaatgcagtcaaaacacccatacacataaaactaa

agtaattaaccagaaagaaaaaaagtcacatttgtgttgaacatggtagcacacacctttaattctatctagcacatgga

agtcagagcagatagatctctgtgagtttgaggccagtctggtttacatagtgaattccattccagctagggctaaatag

ttagaccttgcctttaaaagaaaaaaaaaaagtgagatttaagcaagtcctagtggcacacacgtgcagttctagctact

aagaaggctggaatatttgcacaaccaggaattcaagtccagcctaggtagcatagcaagaccctgtctgtcctacgtag

atttctattgttgtgatgaccaaaagctacttgggaatttatttcagcttacagttgaaatgcatcatgaaggaaagtca

gaacaggaattcgaggcaggaacctggaggccagaacttaagcacaaaccatagaggaactgcttactggcttgctcagc

ttgctttcttatgcatccctgaatgacctacccgggaatggcaccatccacagtaaactgggctctttcacatcaagcac

caatcaagggtctgtttaatccagcgcttctggtttgatgtcctcctgcagggctagagatgcaaggagcttattgtatc

acagtgcgtatgggtctgtcctcagtttcagtaataaatactttaaatttatcagggtggtttactctgggctctaatgt

gatcttatctccaaagaaaaacaaatcattctatttcctttaagaaagcctcatacgccttccgtttctttgagattcac

aatctgatgccgtggtttgggcctcccaccctttcctgtaacagcactttcttccatccctgtttcaaactctttcctta

caggattcggcttagttctccccgagctaacctctggggtggaggttgcaagatcttgacgccagggtccgctcttgcac

tccgtttcagtagccagggggaggacctggcaagcagggtgtgctgccacggcggccacagcctctggaatgcagctgctaggacctcttatgccagtagagcctgaactgcagaatcgctgcccctccaaagcagctggacccactaacctttaaaggt

cttggagttgcatccgtcgctctccactaaattcggcctctaccctttccaatcccttctacctacccatacagagtgag

attcctctgatgtaaccaagtagccagagatcatcctcccgattattttctttcttagccccctccccttcctctccgtc

tctcggtccttttagctcaagatggtggccctccttagctctgttccatctcctccagcacctcccttccagatttccta

cacactcaagccaggggttaacctgctggagttgacccaagttccattctgccccgcccctccaccctccctagggcttg

tccttggatcctccccttcccgccccctcctccagttccatccttctttgctctctttgcccttcccccttcccgcccct

gtccactccaacctgctggcccctcccgggcctgagccctgggcccgcctcttctaacttaggactcctgcgatccccaa

ctccgcactgccaagcttggcattccggtactgttggtaaagccaccatggaagacgccaaaaacataaagaaaggcccg

gcgccattctatccgctggaagatggaaccgctggagagcaactgcataaggctatgaagagatacgccctggttcctgg

aacaattgcttttacagatgcacatatcgaggtggacatcacttacgctgagtacttcgaaatgtccgttcggttggcag

aagctatgaaacgatatgggctgaatacaaatcacagaatcgtcgtatgcagtgaaaactctcttcaattctttatgccg

gtgttgggcgcgttatttatcggagttgcagttgcgcccgcgaacgacatttataatgaacgtgaattgctcaacagtat

gggcatttcgcagcctaccgtggtgttcgtttccaaaaaggggttgcaaaaaattttgaacgtgcaaaaaaagctcccaa

tcatccaaaaaattattatcatggattctaaaacggattaccagggatttcagtcgatgtacacgttcgtcacatctcat

ctacctcccggttttaatgaatacgattttgtgccagagtccttcgatagggacaagacaattgcactgatcatgaactc

ctctggatctactggtctgcctaaaggtgtcgctctgcctcatagaactgcctgcgtgagattctcgcatgccagagatc

ctatttttggcaatcaaatcattccggatactgcgattttaagtgttgttccattccatcacggttttggaatgtttact

acactcggatatttgatatgtggatttcgagtcgtcttaatgtatagatttgaagaagagctgtttctgaggagccttca

ggattacaagattcaaagtgcgctgctggtgccaaccctattctccttcttcgccaaaagcactctgattgacaaatacg

atttatctaatttacacgaaattgcttctggtggcgctcccctctctaaggaagtcggggaagcggttgccaagaggttc

catctgccaggtatcaggcaaggatatgggctcactgagactacatcagctattctgattacacccgagggggatgataa

accgggcgcggtcggtaaagttgttccattttttgaagcgaaggttgtggatctggataccgggaaaacgctgggcgtta

atcaaagaggcgaactgtgtgtgagaggtcctatgattatgtccggttatgtaaacaatccggaagcgaccaacgccttg

attgacaaggatggatggctacattctggagacatagcttactgggacgaagacgaacacttcttcatcgttgaccgcct

gaagtctctgattaagtacaaaggctatcaggtggctcccgctgaattggaatccatcttgctccaacaccccaacatct

tcgacgcaggtgtcgcaggtcttcccgacgatgacgccggtgaacttcccgccgccgttgttgttttggagcacggaaag

acgatgacggaaaaagagatcgtggattacgtcgccagtcaagtaacaaccgcgaaaaagttgcgcggaggagttgtgtt

tgtggacgaagtaccgaaaggtcttaccggaaaactcgacgcaagaaaaatcagagagatcctcataaaggccaagaagggcggaaagatcgccgtgtaattctagagtcggggcggccggccgcttcgagcagacatgataagatacattgatgagttt

ggacaaaccacaactagaatgcagtgaaaaaaatgctttatttgtgaaatttgtgatgctattgctttatttgtaaccat

tataagctgcaataaacaagttaacaacaacaattgcattcattttatgtttcaggttcagggggaggtgtgggaggttt

tttaaagcaagtaaaacctctacaaatgtggtaaaatcgataaggatccgtcgaccgatgcccttgagagccttcaaccc

agtcagctccttccggtgggcgcggggcatgactatcgtcgccgcacttatgactgtcttctttatcatgcaactcgtag

gacaggtgccggcagcgctcttccgcttcctcgctcactgactcgctgcgctcggtcgttcggctgcggcgagcggtatc

agctcactcaaaggcggtaatacggttatccacagaatcaggggataacgcaggaaagaacatgtgagcaaaaggccagcaaaaggccaggaaccgtaaaaaggccgcgttgctggcgtttttccataggctccgcccccctgacgagcatcacaaaaatcgacgctcaagtcagaggtggcgaaacccgacaggactataaagataccaggcgtttccccctggaagctccctcgtgcgctctcctgttccgaccctgccgcttaccggatacctgtccgcctttctcccttcgggaagcgtggcgctttctcatagct

cacgctgtaggtatctcagttcggtgtaggtcgttcgctccaagctgggctgtgtgcacgaaccccccgttcagcccgac

cgctgcgccttatccggtaactatcgtcttgagtccaacccggtaagacacgacttatcgccactggcagcagccactgg

taacaggattagcagagcgaggtatgtaggcggtgctacagagttcttgaagtggtggcctaactacggctacactagaa

gaacagtatttggtatctgcgctctgctgaagccagttaccttcggaaaaagagttggtagctcttgatccggcaaacaa

accaccgctggtagcggtggtttttttgtttgcaagcagcagattacgcgcagaaaaaaaggatctcaagaagatccttt

gatcttttctacggggtctgacgctcagtggaacgaaaactcacgttaagggattttggtcatgagattatcaaaaagga

tcttcacctagatccttttaaattaaaaatgaagttttaaatcaatctaaagtatatatgagtaaacttggtctgacagt

taccaatgcttaatcagtgaggcacctatctcagcgatctgtctatttcgttcatccatagttgcctgactccccgtcgt

gtagataactacgatacgggagggcttaccatctggccccagtgctgcaatgataccgcgagacccacgctcaccggctc

cagatttatcagcaataaaccagccagccggaagggccgagcgcagaagtggtcctgcaactttatccgcctccatccag

tctattaattgttgccgggaagctagagtaagtagttcgccagttaatagtttgcgcaacgttgttgccattgctacagg

catcgtggtgtcacgctcgtcgtttggtatggcttcattcagctccggttcccaacgatcaaggcgagttacatgatccc

ccatgttgtgcaaaaaagcggttagctccttcggtcctccgatcgttgtcagaagtaagttggccgcagtgttatcactc

atggttatggcagcactgcataattctcttactgtcatgccatccgtaagatgcttttctgtgactggtgagtactcaac

caagtcattctgagaatagtgtatgcggcgaccgagttgctcttgcccggcgtcaatacgggataataccgcgccacata

gcagaactttaaaagtgctcatcattggaaaacgttcttcggggcgaaaactctcaaggatcttaccgctgttgagatcc

agttcgatgtaacccactcgtgcacccaactgatcttcagcatcttttactttcaccagcgtttctgggtgagcaaaaac

aggaaggcaaaatgccgcaaaaaagggaataagggcgacacggaaatgttgaatactcatactcttcctttttcaatatt

attgaagcatttatcagggttattgtctcatgagcggatacatatttgaatgtatttagaaaaataaacaaataggggtt

ccgcgcacatttccccgaaaagtgccacctgacgcgccctgtagcggcgcattaagcgcggcgggtgtggtggttacgcgcagcgtgaccgctacacttgccagcgccctagcgcccgctcctttcgctttcttcccttcctttctcgccacgttcgccg

gctttccccgtcaagctctaaatcgggggctccctttagggttccgatttagtgctttacggcacctcgaccccaaaaaa

cttgattagggtgatggttcacgtagtgggccatcgccctgatagacggtttttcgccctttgacgttggagtccacgtt

ctttaatagtggactcttgttccaaactggaacaacactcaaccctatctcggtctattcttttgatttataagggattt

tgccgatttcggcctattggttaaaaaatgagctgatttaacaaaaatttaacgcgaattttaacaaaatattaacgctt

acaatttgccattcgccattcaggctgcgcaactgttgggaagggcgatcggtgcgggcctcttcgctattacgccagcc

caagctaccatgataagtaagtaatattaaggtacgggaggtacttggagcggccgcaataaaatatctttattttcatt

acatctgtgtgttggttttttgtgtgaatcgatagtactaacatacgctctccatcaaaacaaaacgaaacaaaacaaac

tagcaaaataggctgtccccagtgcaagtgcaggtgccagaacatttctctatcgata

**Figure S1. Extraction and characterization of MSC-Exos**. **A**) Schematic illustration of the MSC-Exo extraction by ultracentrifugation and size exclusion chromatography. **B**) Representative TEM image of MSC-Exos. Scale bar: 50 nm. **C**) NTA of MSC-Exos, showing particle size distribution and concentration. **D**) WB of MSC-Exo surface markers and negative control.


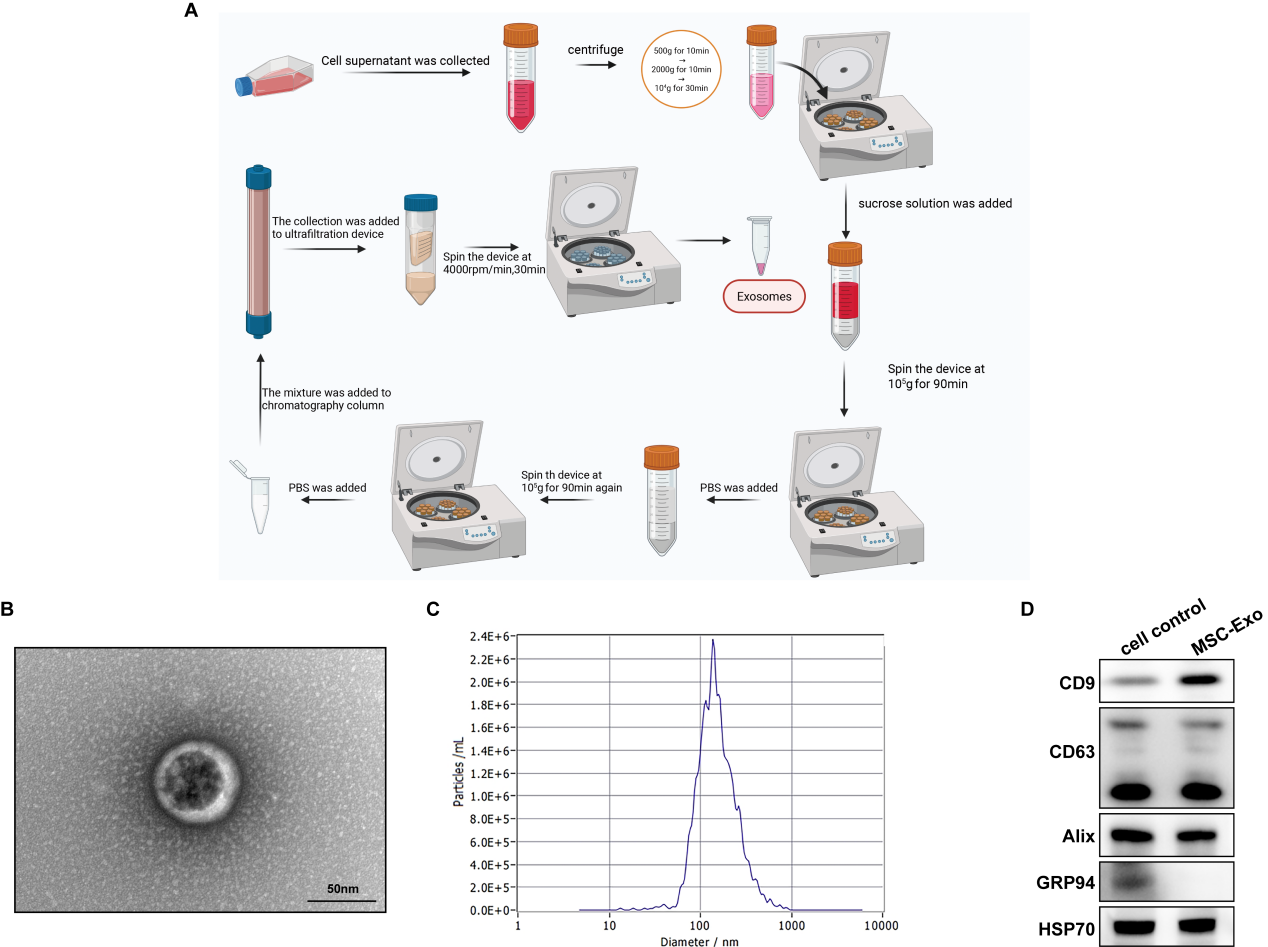


**Figure S2.** **A**) TUNEL staining of H₂O₂-induced apoptotic AML12 cells. Magnification: 20×; scale bar: 50 μm. **B**) Standard curve for let-7a-5p quantification by qPCR. The linear regression equation and coefficient of determination are shown. **C**) Comparison of CT values between MSC-Exo^let-7a-5p^ and MSC-Exo^control^ groups post-electroporation.****p < 0.001.*


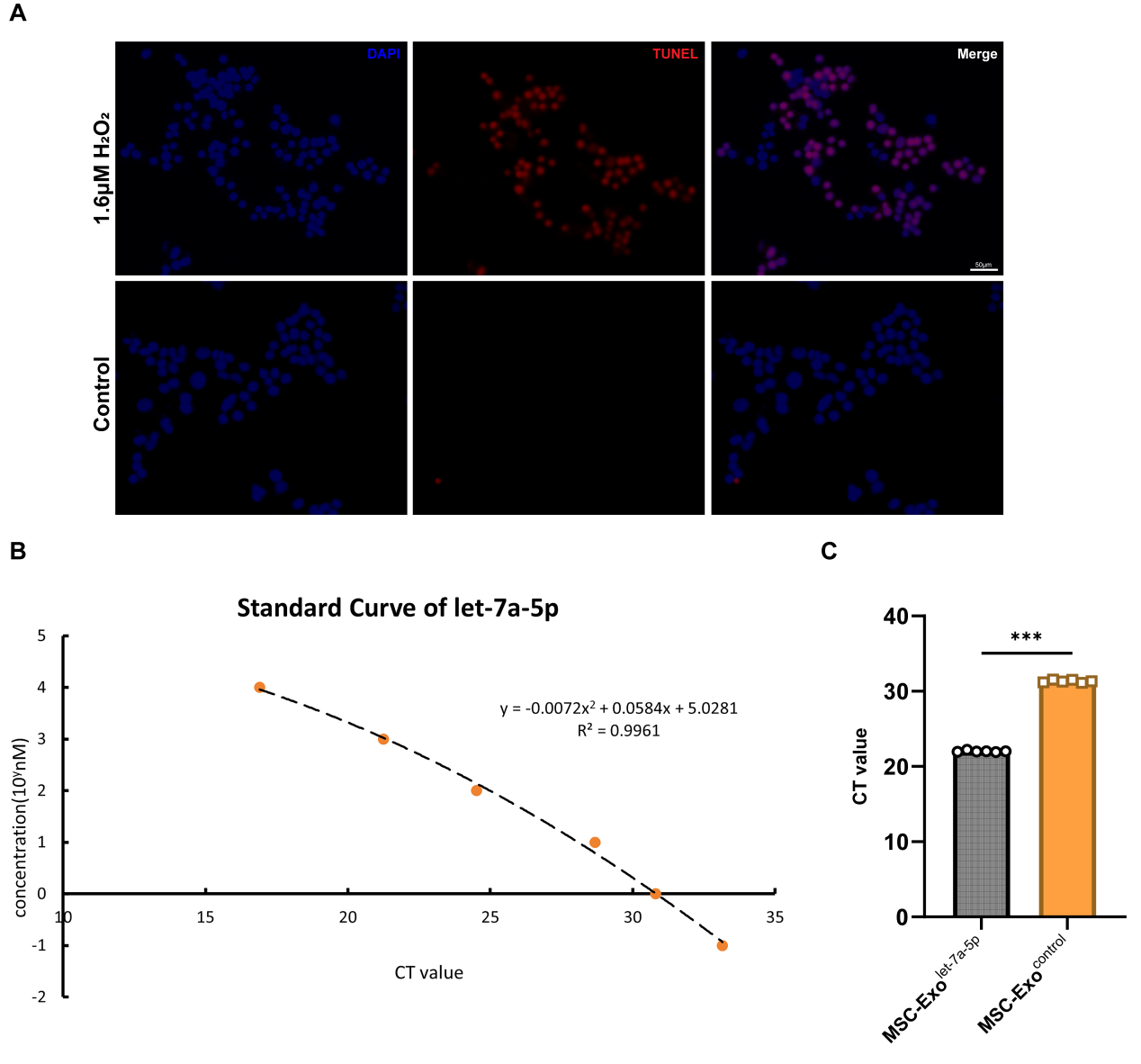

Supplement: szaf058_Supplementary_Data [file szaf058_supplementary_data.docx]
